# Comparison of methods for calculating conditional expectations of sufficient statistics for continuous time Markov chains

**DOI:** 10.1186/1471-2105-12-465

**Published:** 2011-12-05

**Authors:** Paula Tataru, Asger Hobolth

**Affiliations:** 1 Bioinformatics Research Center, Aarhus University, Aarhus, Denmark

## Abstract

**Background:**

Continuous time Markov chains (CTMCs) is a widely used model for describing the evolution of DNA sequences on the nucleotide, amino acid or codon level. The sufficient statistics for CTMCs are the time spent in a state and the number of changes between any two states. In applications past evolutionary events (exact times and types of changes) are unaccessible and the past must be inferred from DNA sequence data observed in the present.

**Results:**

We describe and implement three algorithms for computing linear combinations of expected values of the sufficient statistics, conditioned on the end-points of the chain, and compare their performance with respect to accuracy and running time. The first algorithm is based on an eigenvalue decomposition of the rate matrix (EVD), the second on uniformization (UNI), and the third on integrals of matrix exponentials (EXPM). The implementation in R of the algorithms is available at http://www.birc.au.dk/~paula/.

**Conclusions:**

We use two different models to analyze the accuracy and eight experiments to investigate the speed of the three algorithms. We find that they have similar accuracy and that EXPM is the slowest method. Furthermore we find that UNI is usually faster than EVD.

## Background

In this paper we consider the problem of calculating the expected time spent in a state and the expected number of jumps between any two states in discretely observed continuous time Markov chains (CTMCs). The case where the CTMC is only recorded at discretely observed time points arises in molecular evolution where DNA sequence data is extracted at present day and past evolutionary events are missing. In this situation, efficient methods for calculating these types of expectations are needed. In particular, two classes of applications can be identified.

The first class of applications is concerned with rate matrix estimation. [1] describes how the expectation-maximization (EM) algorithm can be applied to estimate the rate matrix from DNA sequence data observed in the leaves of an evolutionary tree. The EM algorithm is implemented in the software XRate [2] and has been applied in [3] for estimating empirical codon rate matrices. [1] uses the eigenvalue decomposition of the rate matrix to calculate the expected time spent in a state and the expected number of jumps between states.

The second class of applications is concerned with understanding and testing various aspects of evolutionary trajectories. In [4] it is emphasized that analytical results for jump numbers are superior to simulation approaches and various applications of jump number statistics are provided, including a test for the hypothesis that a trait changed its state no more than once in its evolutionary history and a diagnostic tool to measure discrepancies between the data and the model. [4] assumes that the rate matrix is diagonalizable and that the eigenvalues are real, and applies a spectral representation of the transition probability matrix to obtain the expected number of state changes.

[5] and [6] describe a method, termed substitution mapping, for detecting coevolution of evolutionary traits, and a similar method is described in [7]. The substitution mapping method is based on the expected number of substitutions while [7] base their statistics on the expected time spent in a state. Furthermore [7] describes an application concerned with mapping synonymous and non-synonymous mutations on branches of a phylogenetic tree and employs the expected number of changes between any two states for this purpose. [8] uses the expected number of state changes to calculate certain labeled evolutionary distances. A labeled evolutionary distance could for example be the number of state changes from or to a specific nucleotide. In [9] substitution mapping is invoked for identifying biochemically constrained sites. In [7] and [8] the summary statistics are calculated using the eigenvalue decomposition method suggested by [1]. In [5,6] and [9] the substitution mapping is achieved using a more direct formula for calculating the number of state changes. In this direct approach an infinite sum must be truncated and it is difficult to control the error associated with the truncation. An alternative is described in [10] where uniformization is applied to obtain the expected number of jumps. [10] uses the expected number of jumps on a branch to detect lineages in a phylogenetic tree that are under selection.

A third algorithm for obtaining the number of changes or time spent in a state is outlined in [11]. The algorithm is based on [12] where a method for calculating integrals of matrix exponentials is described. A natural question arises: which of the three methods (eigenvalue decomposition, uniformization or matrix exponentiation) for calculating conditional expectations of summary statistics for a discretely observed CTMC should be preferred? The aim of this paper is to provide an answer to this question. We describe and compare the three methods. Our implementations in R [13] are available at http://www.birc.au.dk/~paula/. (Furthermore the eigenvalue decomposition and uniformization methods are also available as a C++ class in the bio++ library at http://biopp.univ-montp2.fr/.) The performance and discussion of the algorithms are centered around two applications. The first application is concerned with rate matrix estimation; we estimate the Goldman-Yang codon model [14] using the expectation-maximization algorithm. The second application is based on the labeled distance estimation presented in [8].

Consider a stochastic process {*X*(*s*): 0 ≤ *s *≤ *t*} which can be described by a CTMC with *n *states and an *n *× *n *rate matrix *Q *= (*q*_*cd*_). The off-diagonal entries in *Q *are non-negative and rows sum to zero, i.e. *q*_*cc *_= - Σ_*d*≠*c *_*q*_*cd *_= -*q*_*c*_. Maximum likelihood estimation of the rate matrix from a complete observation of the process is straight forward. The likelihood of the process, conditional on the beginning state *X*(0), is given by (e.g. [15])

(1) $$L\left(Q;\left\{X\left(s\right):0\leq s\leq t\right\}\right)=\exp\left(-\underset{c}{\sum }q_{c}T_{c}\right)\left(\overset{n}{\underset{c=1}{\prod }}\underset{d\neq c}{\prod }q_{cd}^{N_{cd}}\right),$$

where *T*_*c *_is the total time spent in state *c *and *N*_*cd *_is the number of jumps from *c *to *d*. The necessary sufficient statistics for a CTMC are thus the time spent in each state and the number of jumps between any two states. In applications, however, access is limited to DNA data from extant species. The CTMC is discretely observed and we must estimate the mean values of *T*_*c *_and *N*_*cd *_conditional on the end-points *X*(0) = *a *and *X*(*t*) = *b*. From [15] we have that

(2) $$E\left[T_{c}|X\left(0\right)=a,X\left(t\right)=b\right]=E\left[T_{c}|t,a,b\right]=\frac{I_{cc}^{ab}\left(t\right)}{p_{ab}\left(t\right)}$$

(3) $$E\left[N_{cd}|X\left(0\right)=a,X\left(t\right)=b\right]=E\left[N_{cd}|t,a,b\right]=\frac{q_{cd}I_{cd}^{ab}\left(t\right)}{p_{ab}\left(t\right)}$$

where *P*(*t*) = (*p*_*ij*_(*t*)) = *e*^*Qt *^is the transition probability matrix and

(4) $$I_{cd}^{ab}(t)={\int^{\text{​}}}_{0}^{t}p_{ac}(u)p_{db}(t-u)du.$$

Many applications require a linear combination of certain substitutions or times. Examples include the number of transitions, transversions, synonymous and non-synonymous substitutions. In the two applications described below the statistics of interest is a linear combination of certain substitutions and times. Let therefore *C *be an *n *× *n *matrix and denote by Σ(*C*; *t*) the matrix with entries

(5) $$\sum \left(C;a,b,t\right)=\underset{c,d}{\sum }C_{cd}I_{cd}^{ab}\left(t\right).$$

We describe, compare and discuss three methods for calculating Σ(*C*; *t*). The evaluation of the integrals (4) takes *O*(*n*^3^) time and therefore a naive calculation, assuming that *C *contains just one entry different from zero has a *O*(*n*^5^) running time. Even worse, if *C *contains *O*(*n*^2^) entries different from zero, then the naive implementation has a *O*(*n*^7^) running time. For all three methods our implementations of Σ(*C*; *t*) run in *O*(*n*^3^) time.

## Results

### Applications

#### Application 1: Rate matrix estimation

Our first application is the problem of estimating the parameters in a CTMC for evolution of coding DNA sequences which we describe using the 61 × 61 rate matrix (excluding stop codons) given by Goldman and Yang [14]:

(6) $$q_{ij}=\left\{\begin{array}{cc} 0 & \text{if}\;\text{there}\;\text{is}\;\text{more}\;\text{than}\;\text{one}\;\text{difference}\;\text{between}\;\text{codons}\;i\;\text{and}\;j\\ \alpha \kappa \pi_{j} & \text{if}\;j\;\text{is}\;\text{obtained}\;\text{from}\;i\;\text{by}\;\text{a}\;\text{synonymous}\;\text{transition}\\ \alpha \pi_{j} & \text{if}\;j\;\text{is}\;\text{obtained}\;\text{from}\;i\;\text{by}\;\text{a}\;\text{synonymous}\;\text{transversion}\\ \alpha \omega \kappa \pi_{j} & \text{if}\;j\;\text{is}\;\text{obtained}\;\text{from}\;i\;\text{by}\;\text{a}\;\text{non - synonymous}\;\text{transition}\\ \alpha \omega \pi_{j} & \text{if}\;j\;\text{is}\;\text{obtained}\;\text{from}\;i\;\text{by}\;\text{a}\;\text{non - synonymous}\;\text{transversion} \end{array}\right)$$

where *π *is the stationary distribution, *κ *is the transition/transversion rate ratio, *ω *is the non-synonymous/synonymous ratio and *α *is a scaling factor. The stationary distribution *π *is determined directly from the data using the codon frequencies. We estimate the remaining parameters *θ *= (*α*, *κ*, *ω*) using the expectation-maximization (EM) algorithm [16] as described below.

Suppose the complete data **x **is available, consisting of times and types of substitutions in all sites and in all branches of the tree. The complete data log likelihood is, using (1) and (6),

(7) $$\begin{array}{ll} \ell \left(\alpha ,\kappa ,\omega ;x\right) & =-\alpha L_{\text{s,tv}}-\alpha \omega L_{\text{ns,tv}}-\alpha \kappa L_{\text{s,ts}}-\alpha \kappa \omega L_{\text{ns,ts}}\;\\ & \;\;+N\log\alpha +N_{\text{ts}}\log\kappa +N_{\text{ns}}\log\omega ,\;\\ & \; \end{array}$$

where we use the notation

(8) $$L_{\text{s,ts}}=\underset{i}{\sum }T_{i}\underset{j}{\sum }\pi_{j}1\left(\left(i,j\right)\in L_{\text{s,ts}}\right)\;\text{and}\;N_{\text{ts}}=\underset{i,j}{\sum }N_{ij}1\left(\left(i,j\right)\in L_{\text{ts}}\right)$$

where e.g.

$$L_{\text{s},\text{ts}}=\{(i,j):i\;\text{and}\;j\;\text{differ}\;\text{at}\;\text{one}\;\text{position}\;\text{and}\;\text{the}\;\text{substitution}\;\text{of}\;i\;\text{with}\;j\;\text{is}\;\text{a}\;\text{synonymous}\;\text{transition}\}.$$

A similar notation applies for *L*_s,tv_, *L*_ns,ts_, *L*_ns_,_tv_, *N*_ns _and *N*, where the last statistic is the sum of substitutions between all states (*i*, *j*) that differ at one position and s, ns, ts and tv subscripts stand for synonymous, non-synonymous, transition and transversion.

The complete data log likelihood can be maximized easily by making the re-parametrization *β *= *ακ*. We find that

(9) $$\hat{\alpha }=\frac{N_{\text{tv}}}{L_{\text{s,tv}}+\hat{\omega }L_{\text{ns,tv}}},\hat{\beta }=\frac{N_{\text{ts}}}{L_{\text{s,ts}}+\hat{\omega }L_{\text{ns,ts}}}\text{and}\;\hat{\omega }=\frac{-b+\sqrt{b^{2}-4ac}}{2a},$$

where *a *= -*L*_ns,tv_*L*_ns,ts_*N*_s_, *b *= *L*_ns,tv_*L*_s,ts_(*N*_ns _- *N*_tv_) + *L*_ns,ts_*L*_s,tv_(*N*_ns _- *N*_ts_) and *c *= *L*_s,tv_*L*_s,ts_*N*_ns_.

In reality the data **y **is only available in the leaves and the times and types of substitutions in all sites and all branches of the tree are unaccessible. The EM algorithm is an efficient tool for maximum likelihood estimation in problems where the complete data log likelihood is analytically tractable but full information about the data is missing.

The EM algorithm is an iterative procedure consisting of two steps. In the E-step the expected complete log likelihood

(10) $$G\left(\theta ;\theta_{0},y\right)=E_{\theta_{0}}\left[\ell \left(\theta ;x\right)|y\right]$$

conditional on the data **y **and the current estimate of the parameters *θ*_0 _is calculated. In the M-step the parameters are updated by maximizing *G*(*θ*; *θ*_0_,**y**). The parameters converge to a local maximum of the likelihood for the observed data.

The expected log likelihood conditional on the data **y **and under the three parameters *α*, *κ *and *ω *is

(11) $$\begin{array}{ll} E\left[\ell \left(\alpha ,\kappa ,\omega ;x\right)|y\right] & =-\alpha E\left[L_{\text{s,tv}}|y\right]-\alpha \omega E\left[L_{\text{ns,tv}}|y\right]\;\\ & \;-\alpha \kappa E\left[L_{\text{s,ts}}|y\right]-\alpha \kappa \omega E\left[L_{\text{ns,ts}}|y\right]\;\\ & \;+E\left[N|y\right]\text{log}\alpha +E\left[N_{\text{ts}}|y\right]\text{log}\kappa +E\left[N_{ns}|y\right]\text{log}\omega .\;\\ & \; \end{array}$$

Therefore the E-step requires expectations of linear combinations of waiting times in a set of states and number of jumps between certain states. Because of the Markov property this calculation can be divided in two parts. First we use the peeling algorithm [17,18] to obtain the probability $\mathbb{P}\left(\gamma_{k}=a,\beta_{k}=b|y,t_{k}\right)$ that a branch *k *of length *t*_*k *_with nodes *γ*_*k *_and *β*_*k *_above and below the branch, respectively, has end-points *a *and *b*. Second, we calculate the desired summary statistic by summing over all branches. For example we have

(12) $$E\left[L_{\text{s,ts}}|y\right]=\underset{\text{branch}\;k}{\sum }\underset{\;a,b}{\sum }\mathbb{P}\left(\gamma_{k}=a,\beta_{k}=b|y,t_{k}\right)E\left[L_{\text{s,ts}}|t_{k},a,b\right]$$

(13) $$E\left[N_{\text{ts}}|y\right]=\underset{\text{branch}\;k}{\sum }\underset{a,b}{\sum }\mathbb{P}\left(\gamma_{k}=a,\beta_{k}=b|y,t_{k}\right)E\left[N_{\text{ts}}|t_{k},a,b\right].$$

The E-step thus consists of calculating conditional expectations of linear combinations of times such as $E\left[L_{\text{s,ts}}|t_{k},a,b\right]$ and substitutions such as $E\left[N_{\text{ts}}|t_{k},a,b\right]$ where *L*_s,ts _and *N*_ts _are given by (8). In our application *n *= 61 and the first type of statistics $E\left[L_{\text{s,ts}}|t,a,b\right]$ is (up to a factor *p*_*ab*_(*t*)) on the form (5) with diagonal entries $C_{ii}=\sum_{j}\pi_{j}1\left(\left(i,j\right)\in L_{\text{s,ts}}\right)$ and all off diagonal entries equal to zero. The second type of statistics $E\left[N_{\text{ts}}|t,a,b\right]$ is also on the form (5) with off-diagonal entries $C_{ij}=q_{ij}1\left(\left(i,j\right)\in L_{\text{ts}}\right)$ and zeros on the diagonal.

#### Application 2: Robust distance estimation

The second application is a new approach for estimating labeled evolutionary distance, entitled robust counting and introduced in [8]. The purpose is to calculate a distance that is robust to model misspecification. The method is applied to labeled distances, for example, the synonymous distance between two coding DNA sequences. As it is believed that selection mainly acts at the protein level, synonymous substitutions are neutral and phylogenies built on these type of distances are more likely to reveal the true evolutionary history. The distance is calculated using the mean numbers of labeled substitutions conditioned on pairwise site patterns averaged over the empirical distribution of site patterns observed in the data. In the conventional method the average is done over the theoretical distribution of site patterns. The robustness is therefore achieved through the usage of more information from the data and less from the model.

Let *Q *be the rate matrix of the assumed model, *P*(*t*) = *e*^*Qt*^, the labeling be given through a set of pairs ℒ and the data be represented by a pairwise alignment **y **= (**y**_1_, **y**_2_) of length *m*. As data only contains information about the product *Qt*, where *t *is the time distance between the sequences, we can set *t *= 1.

Suppose we observe the complete data consisting of the types of substitutions that occurred in all sites and let $N_{L}=\sum_{i,j}N_{ij}1\left(\left(i,j\right)\in L\right)$ be the labeled number of substitutions. A natural labeled distance is given by $d_{L}=E\left(N_{L}\right)$. The labeled distance is estimated as the average across all sites of the expected number of labeled substitutions conditioned on the observed end points:

(14) $$\begin{array}{ll} {\hat{d}}_{L} & =\frac{1}{m}\overset{m}{\underset{s=1}{\sum }}E\left[N_{L}|X\left(0\right)=y_{1s},X\left(1\right)=y_{2s}\right]\;\\ & =\frac{1}{m}\overset{m}{\underset{s=1}{\sum }}E\left[\underset{i,j}{\sum }N_{i,j}1\left(\left(i,j\right)\in L\right)|1,y_{1s},y_{2s}\right].\; \end{array}$$

Therefore this application requires evaluating a sum on the form (5) with off-diagonal entries $C_{ij}=q_{ij}1\left(\left(i,j\right)\in L\right)$ and zeros on the diagonal.

### Algorithms

The calculation of Σ(*C*; *t*) is based on the integrals $I_{cd}^{ab}\left(t\right)$. In this section we present three existing methods for obtaining the integrals and extend them to obtain Σ(*C*; *t*).

#### Eigenvalue decomposition (EVD)

When the rate matrix *Q *is diagonalizable, the computation of transition probabilities *p*_*ab*_(*t*) and integrals $I_{cd}^{ab}\left(t\right)$ can be done via the eigenvalue decomposition (EVD). EVD is a widely used method for calculating matrix exponentials. Let *Q *= *U*Λ*U*^-1 ^be the eigenvalue decomposition, with Λ = diag(λ_1_, ..., λ_*n*_). It follows that

(15) $$P\left(t\right)=e^{Qt}=e^{\left(U\Lambda U^{-1}\right)t}=Ue^{\Lambda t}U^{-1}.$$

Because Λ is diagonal, *e*^Λ*t *^is also diagonal with ${\left(e^{\Lambda t}\right)}_{ii}=e^{\lambda_{i}t}$.

The integral (4) becomes

(16) $$I_{cd}^{ab}\left(t\right)=\underset{i}{\sum }U_{ai}{\left(U^{-1}\right)}_{ic}\underset{j}{\sum }U_{dj}{\left(U^{-1}\right)}_{jb}J_{ij}\left(t\right)$$

(17) $$\text{where}\;J_{ij}\left(t\right)=\left\{\begin{array}{cc} te^{\lambda_{i}t} & \text{if}\;\lambda_{i}=\lambda_{j}\\ \frac{e^{\lambda_{i}t}-e^{\lambda_{j}t}}{\lambda_{i}-\lambda_{j}} & \text{if}\;\lambda_{i}\neq \lambda_{j}. \end{array}\right)$$

Replacing $I_{cd}^{ab}\left(t\right)$ with (16) in (5), rearranging the sums and using $A_{cj}=\sum_{d}C_{cd}U_{dj},B_{ij}=J_{ij}\left(t\right)\sum_{c}{\left(U^{-1}\right)}_{ic}A_{cj},D_{ib}=\sum_{j}B_{ij}{\left(U^{-1}\right)}_{jb}$ and $\Sigma \left(C;a,b,t\right)=\sum_{i}U_{ai}D_{ib}$ we find

(18) $$\Sigma \left(C;t\right)=U\left[J\left(t\right)\circ \left(U^{-1}CU\right)\right]U^{-1}$$

where ○ represents the entry-wise product.

The eigenvalues and eigenvectors might be complex, but they come in complex conjugate pairs and the final result is always real; for more information we refer to the Supplementary Information in [2]. If the CTMC is reversible, the decomposition can be done on a symmetric matrix obtained from *Q *(e.g. [15]), which is faster and tends to be more robust. Let *π *be the stationary distribution. Due to reversibility, *π*_*a*_*q*_*ab *_= *π*_*b*_*q*_*ba*_, which can be written as Π*Q *= Q*Π where Π = diag(*π*). Let *S *= Π^1/2^*Q*Π^-1/2^.

We have that

(19) $$\begin{array}{ll} S^{*} & =\Pi^{-1∕2}Q^{*}\Pi^{1∕2}=\Pi^{-1∕2}\left(Q^{*}\Pi \right)\Pi^{-1∕2}\;\\ & =\Pi^{-1∕2}\left(\Pi Q\right)\Pi^{-1∕2}=\Pi^{1∕2}Q\Pi^{-1∕2}=S\;\\ & \; \end{array}$$

where S* is the transpose of *S*. Then *S *is symmetric. Let Λ, *V *be its eigenvalues and eigenvectors, respectively. Then *V*Λ*V*^-1 ^= *S *= Π^1/2^*Q*Π^-1/2^, which implies *Q *= (Π^-1/2^*V*)Λ(*V*^-1^Π^1/2^) and it follows that *Q *has the same eigenvalues as *S *and Π^-1/2^*V *for eigenvectors.

The results can be summarized in the following algorithm:

**Algorithm 1**: EVD

**Input**: *Q*, *C*, *t*

**Output**: Σ(*C*; *t*)

Step 1: Determine eigenvalues *λ*_*i*_.

            Determine the eigenvectors *U*_*i *_for *Q *and compute *U*^-1^.

Step 2: Determine matrix *J*(*t*) from (17).

Step 3: Determine matrix Σ(*C*;*t*) from (18).

#### Uniformization (UNI)

The uniformization method was first introduced in [19] for computing the matrix exponential *P*(*t*) = *e*^*Qt*^. In [11] it was shown how this method can be used for calculating summary statistics, even for statistics that cannot be written in integral form. Let *μ *= max_*i *_(*q*_*i*_) and $R=\frac{1}{\mu }Q+I$, where *I *is the identity matrix.

Then

(20) $$P\left(t\right)=e^{\mu \left(R-I\right)t}=\overset{\infty }{\underset{m=0}{\sum }}R^{m}\frac{{\left(\mu t\right)}^{m}}{m!}e^{-\mu t}=\overset{\infty }{\underset{m=0}{\sum }}R^{m}\text{Pois}\left(m;\mu t\right)$$

where Pois(*m*; *λ*) is the probability of *m *occurrences from a Poisson distribution with mean *λ*. Using (20) we also have

(21) $$\begin{array}{ll} I_{cd}^{ab}\left(t\right) & =\int_{0}^{t}p_{ac}\left(u\right)p_{db}\left(t-u\right)du\;\\ & =\int_{0}^{t}\left[\overset{\infty }{\underset{i=0}{\sum }}{\left(R^{i}\right)}_{ac}\frac{{\left(\mu u\right)}^{i}}{i!}e^{-\mu u}\right]\left[\overset{\infty }{\underset{j=0}{\sum }}{\left(R^{j}\right)}_{db}\frac{{\left(\mu \left(t-u\right)\right)}^{j}}{j!}e^{-\mu \left(t-u\right)}\right]du\;\\ & =\overset{\infty }{\underset{i=0}{\sum }}\overset{\infty }{\underset{j=0}{\sum }}{\left(R^{i}\right)}_{ac}{\left(R^{j}\right)}_{db}\frac{\mu^{i+j}}{i!j!}e^{-\mu t}\int_{0}^{t}u^{i}{\left(t-u\right)}^{j}du\;\\ & =\overset{\infty }{\underset{i=0}{\sum }}\overset{\infty }{\underset{j=0}{\sum }}{\left(R^{i}\right)}_{ac}{\left(R^{j}\right)}_{db}\frac{\mu^{i+j}}{i!j!}e^{-\mu t}\frac{i!j!}{\left(i+j+1\right)!}t^{i+j+1}\;\\ & =\frac{1}{\mu }\overset{\infty }{\underset{i=0}{\sum }}\overset{\infty }{\underset{j=0}{\sum }}{\left(R^{i}\right)}_{ac}{\left(R^{j}\right)}_{db}\frac{{\left(\mu t\right)}^{i+j+1}}{\left(i+j+1\right)!}e^{-\mu t}\;\\ & =\frac{1}{\mu }\overset{\infty }{\underset{m=0}{\sum }}\text{Pois}\left(m+1;\mu t\right)\overset{m}{\underset{l=0}{\sum }}{\left(R^{l}\right)}_{ac}{\left(R^{m-l}\right)}_{db}.\;\\ & \; \end{array}$$

Replacing (21) in (5), rearranging the sums and using that $\sum_{d}C_{cd}{\left(R^{m-l}\right)}_{db}={\left(CR^{m-l}\right)}_{cb}$ and $\sum_{c}{\left(R^{l}\right)}_{ac}{\left(CR^{m-l}\right)}_{cb}={\left(R^{l}CR^{m-l}\right)}_{ab}$ we derive

(22) $$\Sigma \left(C;t\right)=\frac{1}{\mu }\overset{\infty }{\underset{m=0}{\sum }}\text{Pois}\left(m+1;\mu t\right)\overset{m}{\underset{l=0}{\sum }}R^{l}CR^{m-l}.$$

The main challenge with this method is the infinite sum and we use (20) to determine a truncation point. In particular if we let *λ *= *μt *and truncate at *s*(*λ*) we can bound the error using the tail of the Poisson distribution:

$$\left|p_{ab}\left(t\right)-\overset{s\left(\lambda \right)}{\underset{m=0}{\sum }}{\left(R^{m}\right)}_{ab}\text{Pois}\left(m;\mu t\right)\right|=\overset{\infty }{\underset{m=s\left(\lambda \right)+1}{\sum }}{\left(R^{m}\right)}_{ab}\text{Pois}\left(m;\mu t\right)\leq \overset{\infty }{\underset{m=s\left(\lambda \right)}{\sum }}\text{Pois}\left(m;\mu t\right).$$

We have that, for large values of *λ*, Pois(λ)≈ℕ(λ,λ), where $\mathbb{N}\left(\mu ,\sigma^{2}\right)$ is the normal distribution with mean *μ *and variance *σ*^2^. Therefore, for large *λ*, the error bound

$$b=\overset{\infty }{\underset{m=s\left(\lambda \right)}{\sum }}\text{Pois}\left(m;\mu t\right)\approx 1-\Phi \left(\frac{s\left(\lambda \right)-\lambda }{\sqrt{\lambda }}\right),$$

where Φ(·) is the cumulative distribution function for the standard normal distribution. Consequently we can approximate the truncation point *s*(*λ*) with $\sqrt{\lambda }\Phi^{-1}\left(1-b\right)+\lambda$. If *b *= 10^-8 ^we obtain Φ^-1 ^(1 - *b*) = 5.6.

**Figure 1:**
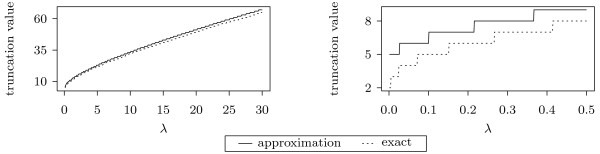
**Poisson truncation**. Comparison between the exact truncation value and the $\left\lceil 4+6\sqrt{\lambda }+\lambda \right\rceil$ approximation. In the plot on the left, *λ *ranges from 0 to 30, while the plot on the right is a zoom-in for values between 0 to 0.5. The plot shows that the approximation is a conservative fit of the Poission tail.

Another way to determine *s*(*λ*) is to use R to evaluate Pois(*m*; *λ*) for values of *m *that gradually increase, until the tail is at most *b *= 10^-8^. Combining these two approaches, we performed a linear regression, approximating the tails from R by $c_{1}+c_{2}\sqrt{\lambda }+c_{3}\lambda$. We obtained *c*_1 _= 4.0731, *c*_2 _= 5.6469, *c*_3 _= 0.9963 but, in order to be conservative, we use $s\left(\lambda \right)=\left\lceil 4+6\sqrt{\lambda }+\lambda \right\rceil$ where ⌈*x*⌉ is the smallest integer greater than or equal to *x*. In Figure 1 we compare the exact truncation value and the linear regression approximation.

The linear regression provides an excellent fit to the tail of the distribution.

In summary we have the following algorithm:

**Algorithm 2**: UNI

**Input**: *Q*, *C*, *t*

**Output**: Σ(*C*; *t*)

Step 1: Determine *μ*, *s*(*μt*) and *R*.

Step 2: Calculate *R*^*m *^for 2 ≤ *m *≤ *s*(*μt*).

Step 3: Calculate $A\left(m\right)=\sum_{l=0}^{m}R^{l}CR^{m-l}$ for 0 ≤ *m *≤ *s*(*μt*).

            using the recursion *A*(*m *+ 1) = *A*(*m*)*R *+ *R*^*m*+1^*C*.

Step 4: Determine Σ(*C*; *t*) from (22).

#### Exponentiation (EXPM)

This method for calculating the integral (4) was developed in [12] and emphasized in [11]. Suppose we want to evaluate $\int_{0}^{t}e^{Qu}Be^{Q\left(t-u\right)}du$, where *Q *and *B *are *n *× *n *matrices. To calculate this integral, we use an auxiliary matrix $A=\left[\begin{array}{cc} Q & B\\ 0 & Q \end{array}\right]$ and the desired integral can be found in the upper right corner of the matrix exponential of *A*:

(23) $$\int_{0}^{t}e^{Qu}Be^{Q\left(t-u\right)}du={\left(e^{At}\right)}_{1:n,\left(n+1\right):2n}.$$

We are interested in

(24) $$\begin{array}{ll} I_{cd}^{ab}\left(t\right) & =\int_{0}^{t}p_{ac}\left(u\right)p_{db}\left(t-u\right)du=\int_{0}^{t}{\left(e^{Qu}\right)}_{ac}{\left(e^{Q\left(t-u\right)}\right)}_{db}du\;\\ & ={\left(\int_{0}^{t}e^{Qu}1_{\left\{\left(c,d\right)\right\}}e^{Q\left(t-u\right)}du\right)}_{ab}\;\\ & \; \end{array}$$

where $1_{\left\{\left(c,d\right)\right\}}$ is a matrix with 1 in entry (*c*, *d*) and zero otherwise. We can use this method to determine $I_{cd}^{ab}\left(t\right)$ by simply setting $B=1_{\left\{\left(c,d\right)\right\}}$, construct the auxiliary matrix *A*, calculate the matrix exponential of *At*, and finally read off the integral in entry (*a*, *b*) in the upper right corner of the matrix exponential.

Replacing (24) in (5) and rearranging the terms we have

(25) $$\Sigma \left(C;t\right)=\int_{0}^{t}e^{Qu}\underset{c,d}{\sum }C_{cd}1_{\left\{\left(c,d\right)\right\}}e^{Q\left(t-u\right)}du\;\text{and}\;\underset{c,d}{\sum }C_{cd}1_{\left\{\left(c,d\right)\right\}}=C.$$

Therefore by setting *B *= *C *in the auxiliary matrix we obtain Σ(*C*;*t*).

The EXPM algorithm is as follows:

**Algorithm 3**: EXPM

**Input**: *Q*, *C*, *t*

**Output**: Σ(*C*; *t*)

Step 1: Construct $A=\left[\begin{array}{cc} Q & C\\ 0 & Q \end{array}\right]$.

Step 2: Calculate the matrix exponential *e*^*At*^.

Step 3: Σ(*C*; *t*) is the upper right corner of the matrix exponential.

### Testing

We implemented the presented algorithms in R and tested them with respect to accuracy and speed.

#### Accuracy

The accuracy of the methods depends on the size of the rate matrix and the time *t*. To investigate how these factors influence the result, we used two different CTMCs that allow an analytical expression for (4). The first investigation is based on the Jukes-Cantor model where the rate matrix has uniform rates and variable size *n*:

$$q_{ij}=\left\{\begin{array}{cc} -1 & \text{if}\;i=j\\ \frac{1}{n-1} & \text{if}\;i\neq j. \end{array}\right)$$

*Q *has two unique eigenvalues: 0 with multiplicity 1 and $-\frac{n}{n-1}$ with multiplicity *n*-1. We obtain

$$\begin{array}{c} \;\;\;p_{ij}\left(t\right)=\left\{\begin{array}{cc} \frac{1}{n}+\frac{n-1}{n}\exp\left(-\frac{nt}{n-1}\right) & \text{if}\;i=j\\ \frac{1}{n}-\frac{1}{n}\exp\left(-\frac{nt}{n-1}\right) & \text{if}\;i\neq j \end{array}\right)\\ \text{and}\;I_{cd}^{ab}\left(t\right)=\frac{1}{n^{2}}\left\{\begin{array}{cc} t+t\exp\left(-\frac{nt}{n-1}\right)-\frac{2\left(n-1\right)}{n}\left(1-\exp\left(-\frac{nt}{n-1}\right)\right) & \text{if}\;a\neq c,d\neq b\\ t+{\left(n-1\right)}^{2}t\exp\left(-\frac{nt}{n-1}\right)+\frac{2{\left(n-1\right)}^{2}}{n}\left(1-\exp\left(-\frac{nt}{n-1}\right)\right) & \text{if}\;a=c,d=b\\ t-\left(n-1\right)t\exp\left(-\frac{nt}{n-1}\right)+\frac{\left(n-2\right)\left(n-1\right)}{n}\left(1-\exp\left(-\frac{nt}{n-1}\right)\right) & \text{otherwise}\text{.} \end{array}\right) \end{array}$$

**Figure 2:**
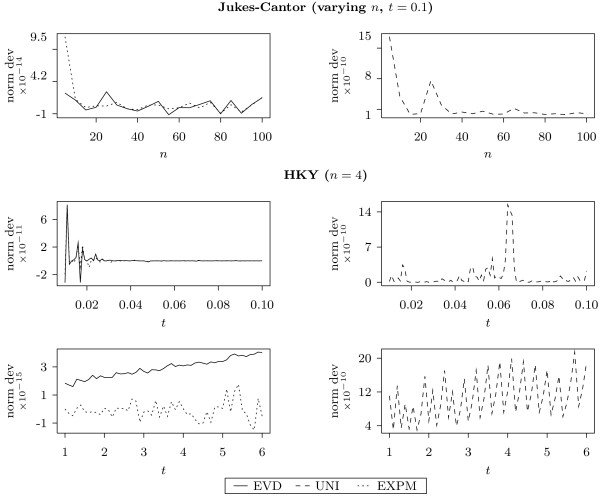
**Accuracy results**. Accuracy has been tested using JC and HKY models. For each run, the normalized deviation is calculated: $\left(\hat{\Sigma }\left(C;a,b,t\right)-\Sigma \left(C;a,b,t\right)\right)∕\Sigma \left(C;a,b,t\right)$ where Σ is the correct value and $\hat{\Sigma }$ is the calculated one. Each plotted point represents the average over *a*, *b *and 5 different randomly generated matrices *C *as described in the main text.

We compared the result from all three methods against the true value of (5) for size *n *ranging from 5 to 100, *t *= 0.1 and random binary matrices *C*. Entries in *C *are 1 with probability $\frac{1}{2}$. For each fixed size, we generated 5 different matrices *C*. The average normalized deviation is shown in Figure 2.

The second CTMC is the HKY model:

$$Q=\left(\begin{array}{cccc} \cdot & \kappa \pi_{G} & \pi_{C} & \pi_{T}\\ \kappa \pi_{A} & \cdot & \pi_{C} & \pi_{T}\\ \pi_{A} & \pi_{G} & \cdot & \kappa \pi_{T}\\ \pi_{A} & \pi_{G} & \kappa \pi_{C} & \cdot \end{array}\right)$$

where *π *= (0.2,0.2,0.3,0.3) is the stationary distribution and *κ *= 2.15 is the transition/transversion rate ratio. This rate matrix has an analytical result for (4) which can be obtained through the eigenvalue decomposition. The eigenvalues and eigenvectors of *Q *are

$$\begin{array}{c} \;\lambda =\left(0,-1,-\pi_{Y}\kappa -\pi_{R},-\pi_{R}\kappa -\pi_{Y}\right)\;\;\text{where}\;\pi_{R}=\pi_{A}+\pi_{G}\;\text{and}\;\pi_{Y}=\pi_{C}+\pi_{T},\\ U=\left(\begin{array}{cccc} 1 & -\frac{\pi_{Y}}{\pi_{R}} & 0 & -\frac{\pi_{G}}{\pi_{A}}\\ 1 & -\frac{\pi_{Y}}{\pi_{R}} & 0 & 1\\ 1 & 1 & -\frac{\pi_{T}}{\pi_{C}} & 0\\ 1 & 1 & 1 & 0 \end{array}\right),\;\;\;\;\;\;U^{-1}=\left(\begin{array}{cccc} \pi_{A} & \pi_{G} & \pi_{C} & \pi_{T}\\ -\pi_{A} & -\pi_{G} & \frac{\pi_{C}\pi_{R}}{\pi_{Y}} & \frac{\pi_{T}\pi_{R}}{\pi_{Y}}\\ 0 & 0 & -\frac{\pi_{C}}{\pi_{Y}} & \frac{\pi_{C}}{\pi_{Y}}\\ -\frac{\pi_{A}}{\pi_{R}} & \frac{\pi_{A}}{\pi_{R}} & 0 & 0 \end{array}\right). \end{array}$$

From this, using the symbolic operations in Matlab [20], we obtained the final analytic expression for (4). Using this model we compared for all three methods the true value of (5) for various values of *t *and randomly generated binary matrices *C*. For each *t *we generated 5 different matrices *C*. The average normalized deviation is shown in Figure 2.

In both cases, all methods showed good accuracy as the normalized deviation was no bigger than 3 × 10^-9^. We also note that EXPM tended to be the most precise while UNI provided the worst approximation. To further investigate the accuracy, we performed calculations on randomly generated reversible rate matrices: we first obtained the stationary distribution from the Dirichlet distribution with shape parameters equal to 1, then all entries *q*_*ij *_with *i *≥ *j *from the exponential distribution with parameter 1 and finally calculated the remaining entries using the reversibility property. In all the runs the relative difference between EVD, UNI and EXPM was less than 10^-5^. This indicated that all three methods have a similar performance in a wide range of applications.

#### Speed

##### Partition of computation

Assume we need to evaluate Σ(*C*; *t*) for a fixed matrix *C *and multiple time points *t *∈ {*t*_1_,...*t*_*k*_}. In each iteration of the EM-algorithm in Application 1 we need this type of calculation while in order to calculate the labeled distance in Application 2 just one time point is required. Using EVD (Algorithm 1) we do the eigenvalue decomposition (Step 1) once and then, for each time point *t*_*i*_, we apply Step 2 and Step 3. The eigenvalue decomposition, achieved through the R function *eigen*, has a running time of *O*(*n*^3^). In Step 2 we determine *J*(*t*) and this takes *O*(*n*^2^) time. Step 3 has a running time of *O*(*n*^3^) due to the matrix multiplications.

If instead we apply UNI (Algorithm 2), we run Steps 1-3 for the largest time point max(*t*_*i*_) and then, for each time point *t*_*i*_, we apply Step 4. Steps 1-3 take *O *(*s*(*μ*max (*t*_*i*_)) *n*^3^) time, and Step 4 takes *O*(*s*(*μt*_*i*_)*n*^2^) time for each *i *∈ {1,..., *k*}. Therefore, even though the total time for both methods is *O*(*n*^3^), the addition of one time point contributes with *O*(*n*^3^) for EVD, but only *O*(*s*(*μt*)*n*^2^) for UNI. Recall that the constant *s*(*μt*) is the truncation point for the infinite sum in the uniformization method.

In the case of EXPM (Algorithm 3) we need to calculate the matrix exponential at every single time point. We used the *expm *R package [21] with the *Higham08 *method. This is a Padé approximation combined with an improved scaling and squaring [22] and balancing [23]. The running time is *O*(*n*^3^).

Table 1 provides an overview of the running times for each of the methods. The algorithms are divided into precomputation and main computation where the precomputation consists of steps that must be executed only once, while in the main computation we calculate the value of Σ(*C*;*t*) for every time point under consideration.

**Table 1:** Running time complexity

Method	EVD	UNI	EXPM
Precomputation			
Steps	1	1-3	none
Order	*O*(*n*^3^)	*O*(*s*(*μt*)*n*^3^)	

Main Computation			
Steps	2-3	4	1-3
Order	*O*(*n*^3^)	*O*(*s*(*μt*)*n*^2^)	*O*(*n*^3^)

##### Experiments

We tested the speed of the algorithms in six experiments based on the presented applications and two more experiments using a non-reversible matrix.

##### GY

The first experiment corresponded to running the EM algorithm on real data consisting of DNA sequences from the HIV *pol *gene described in [24]. HIV has been extensively studied with respect to selection pressure and drug resistance and in [24] the authors document convergent evolution in *pol *gene caused by drug resistance mutations. The observed data y was a multiple codon alignment of the sequences. For simplicity, we did not consider the columns with gaps or ambiguous nucleotides. To compare the performance of the methods as a function of the size of the data set, we applied the EM algorithm for 15 data sets containing from 2 up to 16 sequences each, extracted from the HIV *pol *gene data. For each set we assumed the sequences were related according to a fixed tree; we have reconstructed the phylogenies in Mega [25] using the Jukes-Cantor model and Neighbor-Joining. We ran the EM algorithm until all three parameters converged. Experiments two and three used the previously estimated matrix *Q *given by (6) with *α *= 10.5, *κ *= 4.27 and *ω *= 0.6. We let *C*_*ij *_= *q*_*ij *_and *C*_ii _= 0, corresponding to calculating the total number of expected substitutions E[N|t,a,b], and computed the value of Σ(*C*; *t*_*k*_) for 10 equidistant sorted time points *t*_*k *_with 1 ≤ *k *≤ 10 (Table 2).

**Table 2:** Experimental design

GY						
**Experiment**		**2**			**3**	
***k***	***t*_*k*_**	***μt*_*k*_**	***s*(*μ*_*tk*_)**	***t*_*k*_**	***μt*_*k*_**	***s*(*μ*_*tk*_)**

1	0.0017	0.0045	5	0.1	0.2668	8
2	0.0032	0.0085	5	0.2	0.5337	9
3	0.0046	0.0124	5	0.3	0.8005	11
4	0.0061	0.0163	5	0.4	1.0674	12
5	0.0076	0.0202	5	0.5	1.3342	13
6	0.0090	0.0241	5	0.6	1.6010	14
7	0.0105	0.0281	6	0.7	1.8679	15
8	0.0120	0.0320	6	0.8	2.1347	15
9	0.0135	0.0359	6	0.9	2.4015	16
10	0.0150	0.0398	6	1.0	2.6684	17

GTR						

Experiment		5			6	
*k*	*t*_*k*_	*μt*_*k*_	*s*(*μt*_*k*_)	*t*_*k*_	*μt*_*k*_	*s*(*μt*_*k*_)

1	0.1760	0.2668	8	0.1	0.1516	7
2	0.3520	0.5337	9	0.6	0.9098	11
3	0.5280	0.8005	11	1.1	1.6680	14
4	0.7039	1.0674	12	1.6	2.4262	16
5	0.8798	1.3342	13	2.1	3.1844	18
6	1.0558	1.6010	14	2.6	3.9426	20
7	1.2318	1.8679	15	3.1	4.7008	22
8	1.4077	2.1347	15	3.6	5.4590	24
9	1.5837	2.4015	16	4.1	6.2172	26
10	1.7597	2.6684	17	4.6	6.9754	27

UNR						

Experiment		7			8	
*k*	*t*_*k*_	*μt*_*k*_	*s*(*μ*_*tk*_)	*t*_*k*_	*μt*_*k*_	*s*(*μ*_*tk*_)

1	0.0379	0.1516	7	0.1	0.4	9
2	0.2275	0.9098	11	0.6	2.4	16
3	0.4170	1.6680	14	1.1	4.4	21
4	0.6066	2.4262	16	1.6	6.4	26
5	0.7961	3.1844	18	2.1	8.4	30
6	0.9857	3.9426	20	2.6	10.4	34
7	1.1752	4.7008	22	3.1	12.4	38
8	1.3648	5.4590	24	3.6	14.4	42
9	1.5543	6.2172	26	4.1	16.4	45
10	1.7439	6.9754	27	4.6	18.4	49

The table shows the time points *t*_*k*_, *μ*_*tk *_and the approximation of the Poission tail *s*(*μt*_*k*_). For experiment 2, *t*_*k *_spanned the interval that contains the 10 longest branch lengths from the phylogeny of the 16 HIV *pol *sequences. In experiment 3 we started at 0.1 and ended at 1. We wished to design experiment 5 such that the corresponding *s*(*μt*_*k*_) was the same as *s*(*μt*_*k*_) from experiment 3. This allowed us to illustrate the relative performance of the methods when running on different sizes of the rate matrix. Experiment 6 was done on time points starting from 0.1 and ending at 4.6. As before, we wished to design experiment 7 such *s*(*μt*_*k*_) corresponded to experiment 6. Experiment 8 used the same *t*_*k *_as experiment 6.

##### GTR

In the fourth experiment we estimated the robust labeled distance of two sequences, using the same set-up as in [8]. For each considered evolutionary distance *t *between 0.1 and 1, we generated 50 pairwise sequence data sets of length 2000 which have evolved for time *t *under the general time reversible (GTR) model with

$$Q=\left(\begin{array}{cccc} \cdot & r_{1}\pi_{G} & r_{2}\pi_{C} & r_{3}\pi_{T}\\ r_{1}\pi_{A} & \cdot & r_{4}\pi_{C} & r_{5}\pi_{T}\\ r_{2}\pi_{A} & r_{4}\pi_{G} & \cdot & r_{6}\pi_{T}\\ r_{3}\pi_{A} & r_{5}\pi_{G} & r_{6}\pi_{C} & \cdot \end{array}\right)$$

where *r *= (0.5, 0.3,0.6, 0.2,0.3, 0.2) and *π *= (0.2, 0.2,0.3, 0.3). For labeling, we considered the jumps to and from nucleotide A, leading to *C*_*ij *_= q_*ij *_if *i *or *j *represents nucleotide A. For each data set, we estimated the GTR parameters as described in [8] and calculated the robust distance. Experiments 5 and 6 used the same GTR matrix and *C*_*ij *_= *q*_*ij *_if *i *or *j *represents nucleotide A and zero otherwise, and computed the value of Σ(*C*;*t*_*k*_) for 10 equidistant sorted time points *t*_*k *_with 1 ≤ *k *≤ 10 (Table 2).

##### UNR

In the last two experiments we used the same set-up as in experiments 5 and 6 but with a different matrix and time points (Table 2). As the speed of EVD is influenced by the type of the model, we decided to employ a non-reversible matrix. We chose the unrestricted model and carefully set the rates such that the matrix has a complex decomposition:

$$Q=\left(\begin{array}{cccc} -4 & 2 & 1 & 1\\ 0 & -3 & 2 & 1\\ 1 & 0 & -3 & 2\\ 2 & 1 & 1 & -4 \end{array}\right).$$

**Figure 3:**
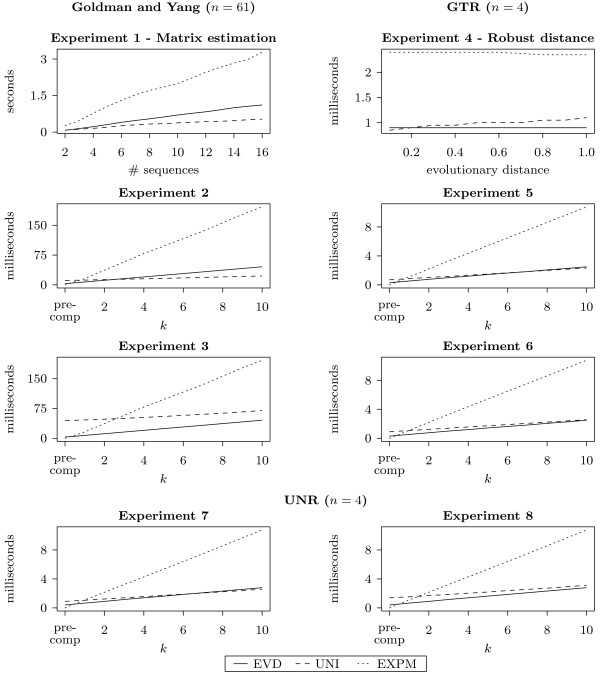
**Experiments results**. Running times for the eight experiments. Experiment 1: rate matrix estimation using EM. The plot shows the running time for calculating the statistics for each method, as a function of the number of sequences included in the data set. For experiments 2 and 3 we calculated the value of Σ(*C*;*t*_*k*_) for 10 time points *t*_*k*_. Each plot starts with the running time of the precomputation and at position *k *we plot the cumulative running time for precomputation and the evaluation of Σ(*C*;*t*_*1*_) for all *t*_*1*_∈ {*t*_*1*_,...,*t*_*k*_}. The values of *t*_*k *_are provided in Table 2. Experiment 4: robust distance estimation. The plot shows the running time for computing the robust distance as a function of the evolutionary distance *t*. Experiments 5-8: similar as for experiments 2 and 3 but with a GTR model (experiments 5 and 6) and UNR model (experiments 7 and 8) instead of a GY model.

Figure 3 shows the results. For experiments 1 and 4, the plots show the recorded running time under each set-up (different number of sequences or different evolutionary distance). For the remaining experiments each plot starts with the running time of the precomputation which, for UNI, is done on the largest time point *t*_10_. Then, at position *k*, we plot the cumulative running time for precomputation and the evaluation of Σ(*C*;*t*_*i*_) for all *i *≤ *k*. Since EVD and EXPM have running times that are independent of *t*_*k*_, the running times for these two algorithms are the same in experiments 2 and 3, 5 and 6, and 7 and 8. Even more, as EXPM is dependent only on the size of the matrix, the running times in experiments 5-8 are the same. We observe that in all our experiments EXPM is the slowest method. Deciding if EVD or UNI is faster depends on the size and type of the matrix, the number of time points and the values of *s*(*μt*). As the main computation for UNI has a running time of *O*(*n*^2^) as opposed to *O*(*n*^3^) for EVD (Table 1), this method should have an increased advantage when the rate matrix is bigger. This means that if many time points are considered, then UNI is generally the faster method. Importantly, we note that the EVD precomputation tends to be faster than the UNI precomputation. We remark that, in the first experiment, UNI proved to be the fastest method while, in the fourth experiment, UNI became slower with the increase of the evolutionary distance between the sequences and it was only faster than EVD for small distances (< 0.2). By setting *t*_*k *_in an appropriate manner (Table 2), we have the same running time for UNI and EXPM for experiment 7 compared to experiment 6. Due to the fact that in experiment 7 we used the UNR matrix, EVD is slower as opposed to experiment 6. In this case, the difference is observable but not very big, but as the size of the matrix increases, this discrepancy increases too. We also note that the difference between the reversible and non-reversible cases is enough to make UNI faster than EVD in the latter case.

## Discussion

The EVD algorithm assumes that the rate matrix is diagonalizable. However, a direct calculation of the integral (4) in the non-diagonalizable case is actually possible using the Jordan normal form for the rate matrix. Let *Q *= *PJP*^-1 ^where *J *is the Jordan normal form of *Q *and *P *consists of the generalized eigenvectors (we recognize that we used *P *and *J *for other quantities earlier but for this discussion this should not cause any confusion and we prefer to use standard notation), i.e. *J *has a block diagonal form *J *= diag(*J*_1_,..., *J*_*κ*_) where *J*_*k *_= *λ*_*k*_*I *+ *N *is a matrix with *λ*_*k *_on the diagonal and 1 on the superdiagonal. We have

(26) $$e^{Qt}=P\text{diag}\left(e^{J_{1}t},\ldots ,e^{J_{K}t}\right)P^{-1},$$

and noting that *N *is a nilpotent matrix with degree *d*_*k *_(equal to the size of block *J*_*k*_) we obtain

(27) $$e^{J_{k}t}=e^{t\lambda_{k}}e^{tN}=e^{\lambda_{k}t}\left(I+tN+\frac{t^{2}}{2}N^{2}+\ldots +\frac{t^{d_{k}-1}}{\left(d_{k}-1\right)!}N^{d_{k}-1}\right).$$

In order to calculate the integral (4) the expressions (26) and (27) are used. It is evident that this procedure is feasible but also requires much bookkeeping.

In [26] an extension of uniformization, adaptive uniformization, is described for calculating transition probabilities in a CTMC. The basic idea is to perform a local uniformization instead of a global uniformization of the rate matrix and thereby have fewer jumps in the jump process. [26] considers a model with rate matrix

$$Q=\left(\begin{array}{cccc} -3v & 3v & 0 & 0\\ \mu & -\left(\mu +2v\right) & 2v & 0\\ 0 & \mu & -\left(\mu +v\right) & v\\ 0 & 0 & 0 & 0 \end{array}\right)$$

(state 4 is an absorbing state). If this process starts in state 1 then the first jump is to state 2 and the second is from state 2 to either state 1 or state 3. This feature can be taken into account by having a so-called adaptive uniformized (AU) jump process where the rate for the first jump is 3*ν*, for the second is *μ *+ 2*ν *and, assuming *μ *+ *ν *> 3*ν*, the rate for the third jump is *μ *+ *ν*. From the third jump the rate in the AU jump process is *μ *+ 2*ν *as in the standard uniformized jump process. The AU jump process has a closed-form expression for the jump probabilities (it is a pure birth process) but is of course more complicated than a Poisson jump process. The advantage is that the AU jump process exhibits fewer jumps. This procedure could very well be useful for codon models where the set of states that the process can be in after one or two jumps are limited because only one nucleotide change is allowed in each state change.

In an application concerned with modeling among-site rate variation, [27] applies the uniformization procedure (20) to calculate the transition probabilities instead of the eigenvalue decomposition method (15). [27] shows, in agreement with our results, that uniformization is a faster computational method than eigenvalue decomposition.

The presented methods are not the only ones for calculating the desired summary statistics. For example, in [5] it is suggested to determine the expected number of jumps from the direct calculation

$$\begin{array}{ll} p_{ab}\left(t\right)E\left[N_{cd}|t,a,b\right] & =\int_{0}^{t}{\left(e^{Qs}\right)}_{ac}q_{cd}{\left(e^{Q\left(t-s\right)}\right)}_{ac}ds\;\\ & =\overset{\infty }{\underset{i=0}{\sum }}\overset{\infty }{\underset{j=0}{\sum }}{\left(Q^{i}\right)}_{ac}q_{cd}{\left(Q^{j}\right)}_{db}\int_{0}^{t}\frac{s^{i}{\left(t-s\right)}^{j}}{i!j!}ds\;\\ & =\overset{\infty }{\underset{k=1}{\sum }}\frac{t^{k}}{k!}\overset{k-1}{\underset{m=0}{\sum }}{\left(Q^{m}\right)}_{ac}q_{cd}{\left(Q^{k-m-1}\right)}_{db},\; \end{array}$$

where the infinite sum is truncated at *k *= 10. The problem with this approach is that it is difficult to bound the error introduced by the truncation. In UNI a similar type of calculation applies but the truncation error can be controlled.

## Conclusion

Recall that EVD assumes that the rate matrix is diagonalizable and this constraint means that EVD is less general than the other two algorithms. We have shown in the Discussion how a direct calculation of the integral (4) is actually still possible but requires much bookkeeping. On top of being less general, EVD is dependent on the type of the matrix: reversible or non-reversible. We have shown how this discrepancy can make EVD slower than UNI even when the state space has size of only 4.

We found that the presented methods have similar accuracy and EXPM is the most accurate one. With respect to running time, it is not straightforward which method is best. We found that both the eigenvalue decomposition (EVD) and uniformization (UNI) are faster than the matrix exponentiation method (EXPM). The main reason for EVD and UNI being faster is that they can be decomposed into a precomputation and a main computation. The precomputation only depends on the rate matrix for EVD while for UNI it also depends on the largest time point and the matrix *C*. We also remark that EXPM involves the exponentiation of a matrix double in size. UNI is particularly fast when the product *μt *is small because in this case only a few terms in the sum (22) are needed.

## Authors' contributions

PT extended the existing methods to linear combinations of statistics, implemented the algorithms and performed the testing. AH conceived the study and guided the development and evaluation of the methods. Both authors wrote the paper. All authors read and approved the final manuscript.
